# Retracted: PASS-Predicted Hepatoprotective Activity of* Caesalpinia sappan* in Thioacetamide-Induced Liver Fibrosis in Rats

**DOI:** 10.1155/2018/7465402

**Published:** 2018-08-12

**Authors:** The Scientific World Journal

The Scientific World Journal has retracted the article titled “PASS-Predicted Hepatoprotective Activity of* Caesalpinia sappan* in Thioacetamide-Induced Liver Fibrosis in Rats” [1]. Figures 3(c) and 3(f) were reused from Figures 1(c) and 1(d) in an article by the same authors, Kadir et al. [2].

An institutional investigation by the University of Malaya found there was no system to index and file data and images to avoid mislabeling and mishandling, which led to errors and duplication of research data. The authors did not thoroughly check the manuscript before submission.

The authors said that both images (1(c) and 1(d)) in Figure 1 in [1] are correct and the same as illustrated in the thesis by Kadir [3]. Panel (c) in both figures represented the same reference group (silymarin group-SY) because they ran two experiments in rats treated with different plants, but with the same reference group. This is also illustrated in Kadir's thesis [3]. Therefore, panel (c) is correct in both Figure 3(c) in [1] and Figure 1(c) in [2]. However, Figure 3(f) was mistakenly uploaded in this article, which probably happened due to incorrect labelling during data collection. This is also illustrated in the thesis [3].

The authors offered to provide replacement figures, but the Editorial Board recommended retraction.
